# Effect of Transmission Setting and Mixed Species Infections on Clinical Measures of Malaria in Malawi

**DOI:** 10.1371/journal.pone.0002775

**Published:** 2008-07-23

**Authors:** Marian C. Bruce, Allan Macheso, Louise A. Kelly-Hope, Standwell Nkhoma, Alex McConnachie, Malcolm E. Molyneux

**Affiliations:** 1 Division of Infection and Immunity, Institute of Biomedical and Life Sciences, Glasgow Biomedical Research Centre, Glasgow University, Glasgow, United Kingdom; 2 Ministry of Health and Population, Government of Malawi, Lilongwe, Malawi; 3 Liverpool School of Tropical Medicine, University of Liverpool, Liverpool, United Kingdom; 4 Malawi-Liverpool-Wellcome Trust Clinical Research Programme, College of Medicine, Blantyre, Malawi; 5 Robertson Centre for Biostatistics, Glasgow University, University Avenue, Glasgow, United Kingdom; London School of Hygiene & Tropical Medicine, United Kingdom

## Abstract

**Background:**

In malaria endemic regions people are commonly infected with multiple species of malaria parasites but the clinical impact of these Plasmodium co-infections is unclear. Differences in transmission seasonality and transmission intensity between endemic regions have been suggested as important factors in determining the effect of multiple species co-infections.

**Principal Findings:**

In order to investigate the impact of multiple-species infections on clinical measures of malaria we carried out a cross-sectional community survey in Malawi, in 2002. We collected clinical and parasitological data from 2918 participants aged >6 months, and applied a questionnaire to measure malaria morbidity. We examined the effect of transmission seasonality and intensity on fever, history of fever, haemoglobin concentration ([Hb]) and parasite density, by comparing three regions: perennial transmission (PT), high intensity seasonal transmission (HIST) and low intensity seasonal transmission (LIST). These regions were defined using multi-level modelling of PCR prevalence data and spatial and geo-climatic measures. The three *Plasmodium* species (*P. falciparum*, *P. malariae* and *P. ovale*) were randomly distributed amongst all children but not adults in the LIST and PT regions. Mean parasite density in children was lower in the HIST compared with the other two regions. Mixed species infections had lower mean parasite density compared with single species infections in the PT region. Fever rates were similar between transmission regions and were unaffected by mixed species infections. A history of fever was associated with single species infections but only in the HIST region. Reduced mean [Hb] and increased anaemia was associated with perennial transmission compared to seasonal transmission. Children with mixed species infections had higher [Hb] in the HIST region.

**Conclusions:**

Our study suggests that the interaction of Plasmodium co-infecting species can have protective effects against some clinical outcomes of malaria but that this is dependent on the seasonality and intensity of malaria transmission.

## Introduction

Infection with multiple *Plasmodium* species is common in malaria endemic regions. It has been suggested that interactions between different species, in conjunction with differences in seasonality and intensity of malaria transmission may underlie variation in the epidemiology and clinical presentation of malaria [1]. Of the four human malaria species, *Plasmodium falciparum* causes the greatest morbidity and mortality but most malaria endemic regions are co-endemic for some or all of the other three human species: *P. malariae*, *P. vivax* and *P. ovale*. Individually, these species cause less severe morbidity and fewer deaths than *P. falciparum* but they are commonly found as co-infections with *P. falciparum* [2], [3]. The effect of multiple species co-infections on the clinical outcomes of malaria is unclear. Most clinical surveys of malaria focus on *P. falciparum* without reference to the potential effects of co-infecting species.

Only a few studies have documented the effects of co-infection on uncomplicated clinical malaria [4]–[9]. Prior or co-infection with *P. malariae* has been implicated in protecting against fevers caused by *P. falciparum* in children in Cote d'Ivoire [4] and in reducing the density of asexual stages and preventing fevers in Tanzanian children [5]. These epidemiological studies of *P. malariae* are supported by experimental data from malaria-therapy infections in humans carried out in the 1940's, in which prior infection with *P. malariae* reduced fever caused by *P. falciparum* [6]. Prior *P. vivax* infection reduced morbidity from subsequent *P. falciparum* infections in Vanuatu [7] and in Papua New Guinea, prior infection with *P. vivax* or *P. malariae* protected against *P. falciparum* fevers [8]. In contrast to these reports of the protective clinical effects of co-infection, an adverse effect has been described in at least one report; carriage of multiple species was associated with greater levels of anaemia than single-species infections in Nigerian children [9]. As well as affecting clinical outcome, interactions between co-infecting species can modify the within-host dynamics of co-infecting malaria parasites [10], [11] and alter the transmission potential of human hosts [12] thereby impacting on the epidemiology of malaria.

In this study we examined the relationship of *Plasmodium* co-infections and clinical measures of malaria under different epidemiological conditions. We compared two districts of Malawi with perennial (Mangochi) and seasonal (Dedza) malaria transmission and examined the effect of transmission intensity by comparing two distinct transmission regions within Dedza District. We used PCR detection of parasites to examine the prevalence and age-distribution of multiple *Plasmodium* species in these diverse regions. In Malawi, as in most of sub-Saharan Africa, the most common sympatric malaria combination is *P. falciparum*, *P. malariae* and *P. ovale*. Previous studies using PCR diagnosis have demonstrated that the prevalence of *P. malariae* and *P. ovale* are greatly underestimated by microscopy especially when these are present in mixed infections with *P. falciparum* [13]–[16]. We investigated the associations between mixed-species infections and fever, history of fever, haemoglobin concentrations and *Plasmodium* densities, to determine how carriage of multiple *Plasmodium* species relates to clinical outcomes of malaria.

## Materials and Methods

### Study sites and population

The study was carried out between March and April 2002 in two districts of Malawi: Dedza in Central Region (14°22′South 34°19′East) and Mangochi in Southern Region (14°28′South 35°15′East). Dedza district lies in a mountainous, semi-forested region at an altitude of >1000 m on the Rift Valley escarpment, bordering Mozambique. Mangochi lies 100 km to the east of Dedza, adjacent to the south western shore of Lake Nyasa, at a lower altitude of approximately 500 m. Annual rainfall is approximately 900 mm in both districts, with the rainy season November–March, during which >90% of rain falls. Dedza temperatures range from 10°C to 28°C, humidity 75% in the wet season and 3°C to 25°C, humidity 60% in the dry season. Annual mean temperature minima and maxima in Dedza are 7°C lower than for Mangochi and wet and dry season humidity values are 77% and 58% respectively [17].

The total population of the two districts at the time of the study was 544,334 for Dedza and 671,102 for Mangochi, within a total Malawi population of 11 million. Outside of the district towns, the populations of both districts reside in dispersed rural settlements where most dwellings are of traditional mud and wood construction with thatched roofs. The predominant occupation in both districts is subsistence farming and in villages adjacent to Lakes Malawi and Malombe, fishing provides additional income. The predominant ethnic group in Dedza is Chewa, who are part of the Nyanja group of Bantu, whilst the population in Mangochi is predominantly of the Yao tribe [18], [19].

Malaria transmission in Dedza district is restricted to the wet season. During the dry season in Dedza, temperature falls below the minimum for development of *Plasmodium* in the vector [20], [21]. *An. arabiensis* and *An. gambiae s.s.* are the major vectors [22]. In contrast, malaria transmission in Mangochi is perennial. Mosquito breeding sites are present throughout the year, owing to the presence of low-lying marshy areas near the lakeshore and the cultivation of rice. The number of infectious bites per person per year has been estimated at 27 but this was made in a year where rainfall in Mangochi was lower than usual [22]. A value of 180 infectious bites per person per year, as measured in other low altitude areas of Malawi, may be more representative [23]. Although transmission is perennial in Mangochi, the transmission intensity varies markedly throughout the year owing to seasonal variation in rainfall, being as much as 6 times greater during the wet season than the dry. The main vectors are *An. gambiae s.l.* and *An. funestus* [22].

Official statistics show that the number of clinical malaria cases and malaria deaths were more than three times greater in Mangochi than in Dedza (annual figures 2004, clinical cases Dedza 116,661, Mangochi 374,995, deaths Dedza 150, Mangochi 498 [24]). Retrospective analysis of paediatric ward admission books from Dedza and Mangochi district hospitals for January–December 2004 (Mangochi n = 1579; Dedza n = 1165) showed that there were also significant differences in the features of malaria-related admissions between the two districts. Children admitted with malaria in Mangochi were younger (Mangochi, mean age 19.4 months, Dedza, 31.9 months, p<0.0001), had more anaemia diagnoses (Mangochi, 39.8%; Dedza, 22.4%, p<0.0001) and had more blood transfusions (Mangochi, 10.3%; Dedza, 4.3%, p<0.0001) than children in Dedza. Children admitted to hospital for malaria in Dedza were older, had more cerebral malaria diagnoses (Dedza, 3.1%; Mangochi, 1.7%, p = 0.016) and had higher hospital case fatality rates (Dedza, 9.6%; Mangochi, 7.3%, p = 0.036) than children admitted in Mangochi (S. Nkhoma, M. Bruce, M. Molyneux, unpublished observations).

**Figure 1:**
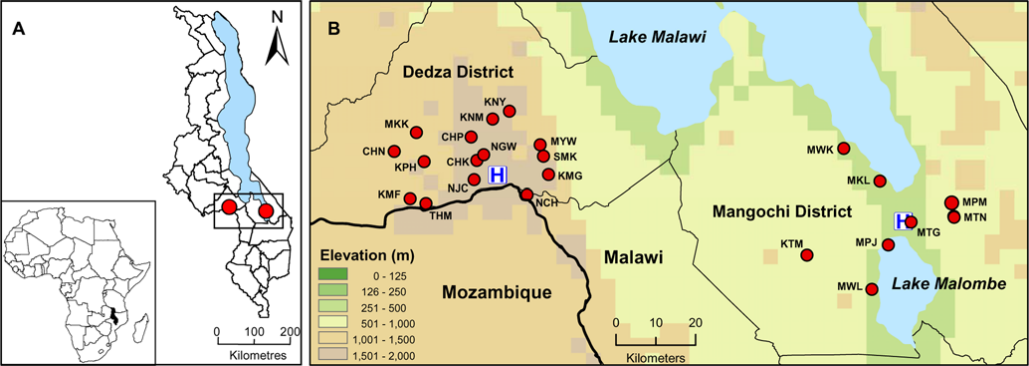
Location of study areas in Dedza and Mangochi districts. (A) relative to the national and district administrative boundaries of Malawi and (B) showing enlargement of study region with position of villages (see Table 3 for 3-letter codes) and district hospitals (marked H).

Participating villages were the largest settlements in randomly selected census enumeration areas (each containing approximately 1000 residents) within a 25 km radius of the district hospitals of Dedza and Mangochi (Figure 1). Sixteen and 8 study villages were selected in Dedza and Mangochi districts, respectively.

### Enrolment and sample collection

Malaria community surveys were carried out between 8 March and 7 April 2002 in Dedza and Mangochi Districts of Malawi. Consenting individuals aged >6 months from each village were enrolled in the study. Oral consent for village participation was obtained from village elders and individual written consent was obtained from adults after explanation of the aims and methods of the study. Written consent for the participation of children was given by their parents or guardians.

From a single finger prick, thick and thin blood smears and a filter paper blood sample for molecular analysis were prepared and haemoglobin concentration was measured using a portable haemoglobinometer (HemoCue, Sweden) according to the manufacturer's instructions. Axillary temperature was measured with a digital thermometer and a questionnaire was administered to collect the following information from each participant: age (years), sex, occupation, religious denomination, history of fever in previous 2 weeks, medicine taken in the previous 2 weeks, overnight stay away from home in previous 4 weeks, use of bednets and other anti-malarial prevention measures.

Following the analysis of blood smears by microscopy, parasite positive participants with axillary temperature ≥37.5°C, or [Hb]≤8.0 g/dl, or ≥5000 parasites/µl were treated within 24 hours of blood sampling with sulphadoxine-pyrimethamine (SP) and ferrous sulphate, according to government guidelines at the time of the study. Participants with overt signs of severe malaria or with haemoglobin≤5.0 g/dl or parasite density ≥100,000 parasites/µl of blood were taken to the district hospital. We did not follow up the clinical outcomes of treated or hospital referred participants. Ethical approval for the study was granted by the National Health Sciences Research Committee, Ministry of Population and Health, Government of Malawi and Glasgow University Ethics Committee for Non-clinical Research Involving Human Subjects.

### Mapping and Remotely Sensed Data

Village location (latitude and longitude) was determined using ground GPS readings (Etrex 12 channel, Garmin Ltd, Kansas, USA) and maps were drawn using ArcInfo 9.1 (Environmental Systems Research Institute, Inc., California, USA). Data on national boundaries, district hospitals, elevation, forested areas, water bodies, marshland and tributaries were obtained from scanned maps [17], datasets and satellite imagery (NASA/ Earthsat 1 km and Global 2′ Elevation Data, ETOPO2) available from Environmental Systems Research Institute, Inc. (ESRI; Redlands, CA) and public websites (http://earth.google.com/; http://www.geographynetwork.com). As no climate data were available, vegetation cover was used as a proxy of greenness (leaf-top photosynthesis) and precipitation availability [25]. Data were obtained from Normalised Difference Vegetation Index (NDVI) values, extracted from MODIS images at 250 m spatial resolution (NASA. http://modis.gsfc.nasa.gov/). Differences between wet and dry seasons were examined and based on values from images 14–29 September 2001 (dry season), NDVI 30 September–15 October 2001 (dry season), NDVI 6–21 March 2002 (wet season), NDVI 22 March–6 April 2002 (wet season).

All epidemiological and environmental data were imported and geo-referenced in the geographical information system software ArcInfo 9.1. Maps were drawn showing each village location and *P. falciparum* prevalence in relation to district hospitals and key environmental characteristics. The following geo-climatic variables were available for each village and extracted for statistical analysis: elevation, latitude, longitude, distance from district hospital, mean dry season NDVI, mean wet season NDVI, and difference between mean wet and dry season NDVI. Additional analysis in relation to elevation was undertaken by visualising topographical/contour differences between each village on a 3D wireframe map created in the surface mapping programme Surfer 7.0 (Golden Software Inc., Golden, CO).

### Detection of Plasmodium species

Primary malaria diagnosis was carried out within 24 hours of blood sampling by microscopic examination of thick and thin blood smears. Blood smears were fixed with methanol (thin smear only) and stained with giemsa. Smears were initially graded for the presence/absence of *Plasmodium* and approximate parasite density, then parasite density counts per 200 leukocytes were made from positive smears. Parasite density per µl of blood was calculated assuming a standard leukocyte count of 8000 per µl of blood. In order to assess the reproducibility of microscopy, a random selection of 10% of smears from each district were re-read.

To confirm microscopy results and detect parasites below the limit of microscopy (around 40 parasites/µl of blood) a retrospective, species-specific PCR [26], [27] was carried out using DNA extracted from filter paper blood samples, as previously described [28]. Samples from 3 villages from each district were tested for all 4 human *Plasmodium* species (*P. falciparum*, *P. vivax*, *P. malariae* and *P. ovale*) whilst the remainder were tested only for *P. falciparum*, *P. malariae* and *P. ovale*. An estimated parasite density of 10 parasites per µl of blood was assigned per species detected by PCR in those samples that were microscopy negative. [The value of 10 parasites per µl is the approximate parasite detection level of PCR. In microscopy negative samples containing all 3 species detected by PCR in the study area (*P. falciparum*, *P. malariae* and *P. ovale*) the use of this estimate results in an density value below the microscopy sensitivity level of 40 parasites per µl]. To assess the reproducibility of PCR detection a random selection of 5% of samples from each district were re-analysed (from the sample DNA extraction) for the presence of *P. falciparum*, *P. malariae* and *P. ovale*.

### Statistical methods

Data were analysed using SPSS v10.0 (Chicago, USA). Data for each district were compared using Pearson's Chi squared test for categorical variables and Mann-Whitney U test for continuous variables. Log_10_ transformation was carried out to normalise parasite density data. Univariate and bivariate regression models (accounting for district and transmission region) were used to determine which variables were associated with the following clinical outcomes measured during community surveys: haemoglobin concentration ([Hb] g/dl), moderate anaemia ([Hb]≤8.0 g/dl) and fever (axillary temperature ≥37.5°C). Linear regression was used for [Hb] and logistic regression for the binary outcomes of anaemia and fever. In the linear regression analyses continuous variables were included as co-variates whilst categorical variables were included as fixed factors.

Mixed effects logistic regression models were fitted for whether or not each individual had PCR-detectable infection, using the statistical software package MIwiN v2.01 (Centre for Multilevel Modelling, University of Bristol, UK). The log odds of being infected was allowed to vary at random between villages and between households within villages, according to a Normal distribution. A series of models was fitted: firstly, without covariates, to estimate the degree of variation between and within villages; secondly, allowing for individual-level fixed effects of sex and age (adult vs. child); thirdly, allowing for village-level fixed effects of elevation above sea level and the mean wet and dry season normalised difference vegetation index (NDVI) in each village; and lastly, allowing for both individual- and village-level fixed effects. Additionally, the final model was refitted allowing for separate random and fixed effects in each district (Dedza or Mangochi). For each model, the variation between villages and between households within villages are reported in terms of the variance in log odds of being infected, and in terms of the estimated odds ratio between units (villages or households) at the 75th and 25th percentiles of the assumed distribution of units. Also reported are the fixed effect estimates, in terms of the associated odds ratio estimates, with 95% confidence intervals. Finally, for the model allowing separate fixed and random effects in each region, p-values are reported for the comparisons of model parameters between villages.

The multiple-kind lottery model was applied to determine if the distribution amongst individuals of *Plasmodium* species (*P. falciparum*, *P. malariae* and *P. ovale*) detected by PCR, differed from an independent random distribution [29]. The following categories were used for analysis of multi-species data: negative, one species, mixed species (ie >1 species). Data for *P. malariae* and *P. falciparum* were also analysed in the absence of the least prevalent species, *P. ovale* (categories: negative, *P. malariae* only, *P. falciparum* only, *P. malariae* and *P. falciparum*). We calculated R_OE Mixed_, R_OE Negative_ or R_OE Pm_ the ratio of observed/expected (and 95% CI) amongst values that deviated from expected mixed, negative or *P. malariae* samples, as a measure of the magnitude of the deviation from randomness.

In order to evaluate all possible associations between clinical measurements and the presence of multiple *Plasmodium* infections in three transmission settings it has been necessary to use multiple testing. Unadjusted p values are reported throughout in order not to obscure possible associations and therefore individual p values should be interpreted with this in mind.

## Results

Clinical measurements and blood samples for parasitological analysis were collected between March and April 2002, from 2918 participants residing in 16 villages within Dedza District and 8 villages within Mangochi District, Malawi (Figure 1). Demographic and malaria data obtained by questionnaire were available from 2840 out of 2918 (97.3%) participants. Table 1 shows a summary of the characteristics of the two study populations. Overall, a greater proportion of females than males were enrolled in both districts (62.0% and 62.7% for Dedza and Mangochi respectively). This resulted from a deficit in the enrolment of adult males, who were absent from households because sampling took place during daylight hours (Table 1). Amongst children, the sex ratio was equitable between districts (% female children, 52.2% and 51.0% for Dedza and Mangochi respectively, p = 0.660) but the proportion of children in the study was greater in Mangochi (54.8%) than in Dedza (48.6%, Table 1). There was a difference in the religious denomination of the two populations. Dedza was predominantly Christian, whilst the majority faith in Mangochi was Islam, reflecting differences in the geopolitical history in the two regions.

**Table 1:** Summary of characteristics of participants living in Dedza and Mangochi Districts.

Population characteristic	Dedza n = 1983		Mangochi n = 935		p value^1^	Odds Ratio (Mangochi/Dedza)	
Female	62.0	(59.9–64.2)	62.7	(59.6–65.8)	0.737	1.02	(0.88–1.21)
Adults	51.4	(49.2–53.6)	45.2	(42.0–48.4)	**0.002**	0.78	(0.67–0.91)
Christian religion	74.3	(72.3–76.2)	26.9	(23.9–29.9)	**<0.0001**	0.13	(0.11–0.15)
Malaria positive by microscopy	23.6	(21.7–25.4)	27.3	(24.4–30.1)	**0.030**	1.22	(1.02–1.45)
Malaria positive by PCR	54.4	(52.2–56.6)	76.7	(74.0–79.4)	**<0.0001**	2.76	(2.32–3.29)
Mean log_10_ parasite density (per µl of blood)	3.0	(2.9–3.1)	3.2	(3.1–3.3)	**0.001**	-	-
Mean log_10_ parasite density including PCR ^2^	1.9	(1.8–1.9)	1.8	(1.7–1.9)	0.354	-	-
P. falciparum positive by PCR	53.2	(51.0–55.4)	75.7	(73.0–78.5)	**<0.0001**	2.74	(2.31–3.26)
P. malariae positive by PCR	7.7	(6.5–8.9)	13.2	(11.0–15.3)	**<0.0001**	1.81	(1.41–2.33)
P. ovale positive by PCR	3.3	(2.5–4.1)	7.8	(6.1–9.5)	**<0.0001**	2.46	(1.75–3.46)
Fever (axillary temp ≥37.5°C)	11.5	(10.1–12.9)	11.9	(9.9–14.0)	0.715	1.05	(0.82–1.33)
Mean haemoglobin concentration (g/dl)	12.0	(11.9–12.1)	10.6	(10.5–10.7)	**<0.0001**	-	-
Mild anaemia (Hb≤11.0 g/dl)	28.7	(26.7–30.7)	55.3	(52.1–58.5)	**<0.0001**	3.07	(2.61–3.61)
Moderate anaemia (Hb≤8.0 g/dl)	3.6	(2.8–4.4)	11.6	(9.5–13.6)	**<0.0001**	3.51	(2.57–4.79)
Treated during study ^3^	18.3	(16.6–20.0)	26.2	(23.4–29.0)	**<0.0001**	1.58	(1.32–1.91)
History of fever in previous 2 weeks	45.8	(43.6–48.0)	42.5	(39.2–45.8)	0.103	0.87	(0.74–1.03)
Taken anti-malarial in previous 2 weeks	7.4	(6.3–8.6)	8.9	(7.0–10.8)	0.172	1.22	(0.92–1.63)
Taken painkiller in previous 2 weeks	21.6	(19.8–23.4)	26.7	(23.8–29.7)	**0.003**	1.32	(1.10–1.60)
Sleeping regularly under a bednet	1.4	(0.9–2.0)	14.2	(11.9–16.6)	**<0.0001**	11.41	(7.50–17.36)
Spent night away in previous 4 weeks	3.1	(2.3–3.8)	2.7	(1.6–3.8)	0.582	0.87	(0.54–1.42)

Values are percentages of total enrolled population. Brackets show 95% confidence intervals. ^1^ Pearson's Chi-square test for categorical variables and Mann-Whitney U test for continuous variables. P values less than 0.05 are in bold. ^2^ Taking into account samples in which *Plasmodium* was detected by PCR but not by microscopy (i.e. present only at sub-microscopic levels), for which density was assumed to be 10 parasites per µl of blood for each species detected. ^3^ People treated with anti-malarials during the study were those with high parasite densities or at greater risk of progression to severe malaria, as defined in *Enrolment and sample collection* section of Materials and Methods.

### Relationship of malaria prevalence with geographical features

**Figure 2:**
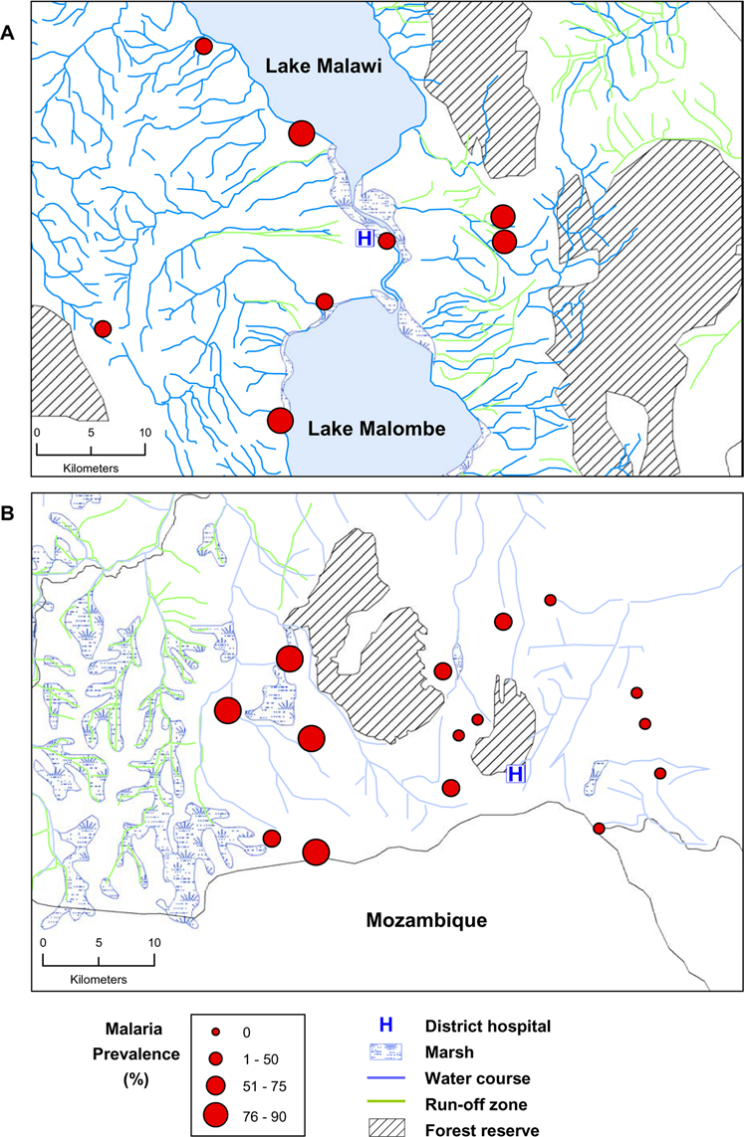
Spatial distribution of malaria prevalence detected by PCR in relation to geographical features in Mangochi (A) and Dedza (B) districts.

**Figure 3:**
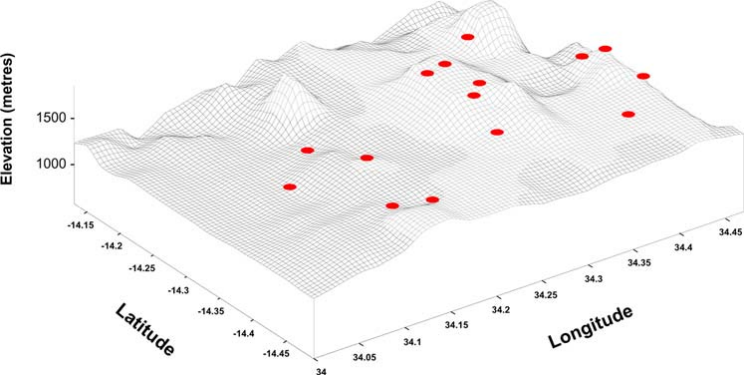
Distribution of villages in Dedza district in relation to 3-dimensional topography and elevation.

The mean prevalence of malaria parasites (any *Plasmodium* species) detected by microscopy in the two districts was remarkably similar during our wet season survey, given the variation in geography and difference in malaria transmission in the two areas. In Dedza, where transmission is seasonal, the mean microscopy prevalence was 23.6% whilst in Mangochi where perennial transmission occurs, prevalence was only slightly higher, 27.3%, a difference significant only at the 0.05 level (Table 1). PCR detection of parasites increased prevalence in Dedza by a factor of 2.3 and in Mangochi by 2.8, giving PCR prevalences of 54.4% and 76.7% respectively (Table 1). Reproducibility of PCR detection was 93.8%. Reproducibility of microscopy was 86.4% for presence/absence of parasites and 79.4% for density grading. The variation in prevalence observed between villages was greater in Dedza than in Mangochi when either microscopy (Dedza: range 2.2–58.4%, standard deviation (S.D.) 13.6; Mangochi: range 15.8–42.3%, S.D. 8.4) or PCR detection was used (Dedza range 17.6–90.3, S.D. 22.4; Mangochi range 65.5–84.6, S.D. 7.7). Examination of the spatial distribution of villages in relation to prevalence showed that in Mangochi, increased prevalence was not associated with proximity to the large water bodies of Lake Malawi or Lake Malombe, nor to marshes or rivers (Figure 2a). However, in Dedza high prevalence was closely associated with proximity to marshy, river run-off areas in the west of the study area, whilst lower prevalence was found in eastern villages located further from the marshes (Figure 2b), and at higher elevation in the east of the study area (Figure 3).

Multi-level regression models were used to investigate the relationship between geo-climatic variables and malaria prevalence, allowing for demographic factors (Table 2). Between-village and between-household variations in prevalence were largely unchanged when adjusting for individual-level effects. Allowing for village-level factors accounted for approximately 25% of the between-village variation, due to a negative association with elevation above sea level. However, between-village variation in prevalence (after accounting for demographic and geo-climatic factors) was observed only in Dedza region, with villages in Mangochi being far more homogeneous. Between-household variation was similar in both regions. There were differences between districts in the effect of elevation and infection probability (p<0.0001), with the odds of infection reducing by about a half for each 100 m elevation in Dedza, but with no corresponding association found in Mangochi. We used remotely sensed normalized difference vegetation index (NDVI) data as a proxy measurement of vegetation greenness and rainfall as this is linked with malaria intensity in some settings in Africa [25]. The mean wet- and dry-season NDVI values for each village showed no association with infection rates.

**Table 2:** Mixed effects models for PCR-detected infection.

			Model 1	Model 2a	Model 2b	Model 3			
						All villages	Mangochi only	Dedza only	p-value Dedza vs. Mangochi
Variance components	Between	σ^2^	1.08	1.16	0.79	0.89	0.05	0.74	**0.026**
	villages	OR_75-25_	4.06	4.28	3.31	3.57	1.37	3.20	
	Between	σ^2^	0.87	0.96	0.82	0.89	0.56	0.90	0.25
	households	OR_75-25_	3.52	3.76	3.40	3.57	2.75	3.59	
Covariate effects	Female vs. Male	OR (95% CI)	-	1.32 (1.07, 1.63)	-	1.31 (1.07, 1.61)	1.36 (0.89, 2.07)	1.34 (1.05, 1.70)	0.95
	Adult vs. Child	OR (95% CI)	-	0.40 (0.32, 0.49)	-	0.41 (0.33, 0.50)	0.15 (0.10, 0.23)	0.60 (0.48, 0.76)	**<0.0001**
	Elevation (/100 m increase)	OR (95% CI)	-	-	0.88 (0.78, 0.99)	0.88 (0.78, 1.00)	1.19 (0.87, 1.63)	0.55 (0.39, 0.77)	**0.0010**
	Mean Dry NDVI (/0.01 increase)	OR (95% CI)	-	-	0.99 (0.83, 1.18)	0.99 (0.82, 1.19)	1.17 (1.02, 1.34)	0.92 (0.72, 1.18)	0.10
	Mean Wet NDVI (/0.01 increase)	OR (95% CI)	-	-	1.01 (0.96, 1.06)	1.01 (0.96, 1.06)	1.00 (0.94, 1.07)	1.02 (0.96, 1.08)	0.68

Model 1: Allowing for random variation between villages and between households; no covariates.

Model 2a: Model 1 plus adjusting for individual effects (age and sex).

Model 2b: Model 1 plus adjusting for village effects (elevation above sea level, mean wet season NDVI and mean dry season NDVI).

Model 3: Model 2 plus adjusting for all covariates.

Estimates of variation between villages and between households (σ^2^−variance of log odds of infection between units, OR_75-25_−odds ratio between units at the 75^th^ and 25^th^ percentiles of distribution) and odds ratios associated with sex, age, elevation above sea level, mean dry NDVI and mean wet NDVI (with 95% confidence intervals) under alternative models. P values less than 0.05 are in bold.

The close association of prevalence with elevation and the spatial relationship of prevalence to geographical features that were likely to provide mosquito breeding sites, led us to conclude that in Dedza, prevalence was an indicator of variation in levels of transmission during the wet season. In order to determine the effect of variation in transmission intensity and transmission seasonality on the distribution and clinical effect of mixed infections, the study population was divided into 3 transmission regions: 1) low intensity seasonal transmission (LIST) (eastern, elevated Dedza villages, n = 10, PCR prevalence = 43.0, 95% CI 40.4–45.6); 2) high intensity seasonal transmission (HIST)(western, low lying Dedza villages, n = 5, PCR prevalence = 84.1, 95% CI 81.6–86.7), 3) perennial transmission (PT) (all Mangochi villages, n = 8, PCR prevalence = 76.7, 95% CI 74.1–79.2, Table 3).

**Table 3:** Summary of parasitological and geographic data per village, ordered within each district by increasing malaria prevalence detected by PCR.

District	Village	Latitude	Longitude	Elevation (metres)	Number of samples	Number of households	Transmission Region^2^	Microscopy Prevalence^3^	PCR Prevalence^4^	% fever^5^	% moderate anaemia^6^	% treated^7^	% bednet usage^8^
Dedza	Kapala^1^				136	52		2.2	17.6	10.3	2.2	11.8	0.7
	Chikanda (CHK)	−14.335	34.288	1751	148	48	LIST	21.6	29.7	11.5	2.0	15.5	0.7
	Simuko (SMK)	−14.326	34.438	1737	110	39	LIST	22.7	30.9	7.3	2.7	11.8	0.0
	Nchochoma (NCH)	−14.412	34.401	1524	138	57	LIST	11.6	34.8	9.4	0.7	12.3	3.7
	Ngwere (NGW)	−14.323	34.303	1769	127	68	LIST	18.1	42.5	13.4	5.5	18.9	0.0
	Muyowe (MYW)	−14.300	34.431	1527	136	48	LIST	17.6	44.1	17.6	1.5	22.1	2.9
	Kanyenda (KNY)	−14.224	34.362	1608	127	59	LIST	25.2	48.0	8.1	2.4	14.2	0.0
	Kamenyagwaza (KMG)	−14.367	34.450	1578	120	37	LIST	19.2	50.8	7.5	1.7	13.3	0.0
	Kanyama (KNM)	−14.242	34.324	1537	101	56	LIST	29.7	58.4	13.9	1.0	19.8	0.0
	Chiphazi (CHP)	−14.283	34.275	1775	142	58	LIST	43.0	58.5	16.9	8.5	28.9	0.7
	Njuchi (NJC)	−14.379	34.281	1578	150	61	LIST	21.3	59.3	10.7	4.7	19.3	0.7
	Kumfunda (KMF)	−14.420	34.137	1467	65	24	HIST	43.1	73.8	15.4	6.2	27.7	0.0
	Makakhula (MKK)	−14.273	34.151	1318	108	39	HIST	13.9	79.6	9.3	4.6	15.7	0.0
	Chinthankwa (CHN)	−14.315	34.101	1303	150	47	HIST	22.7	84.7	6.0	4.7	12.0	9.5
	Kaphala (KPH)	−14.338	34.169	1503	101	39	HIST	58.4	87.1	17.8	6.9	38.6	0.0
	Thambolagwa (THM)	−14.431	34.173	1473	124	58	HIST	24.2	90.3	11.3	3.2	19.4	0.8
	Dedza Total				1983	790		23.6	54.4	11.5	3.6	18.3	14.2
Mangochi	Makawa (MWK)	−14.308	35.117	482	110	47	PT	23.6	65.5	20.9	11.8	30.0	19.1
	Katema (KTM)	−14.547	35.033	886	114	48	PT	15.8	67.5	6.2	12.3	17.5	16.9
	Mtagaluka (MTG)	−14.473	35.269	475	147	45	PT	21.8	72.1	7.6	11.7	23.8	22.3
	Mpinganjira (MPJ)	−14.524	35.217	481	99	31	PT	27.3	75.8	5.1	7.1	17.2	22.8
	Mwalija (MWL)	−14.624	35.180	535	104	38	PT	25.0	79.8	11.5	11.5	24.0	9.8
	Mpamanda (MPM)	−14.462	35.366	535	125	36	PT	27.2	84.0	18.5	9.6	31.2	7.3
	Mkali (MKL)	−14.382	35.198	475	132	44	PT	36.4	84.1	12.2	17.4	34.1	7.3
	Matenganya (MTN)	−14.460	35.365	535	104	39	PT	42.3	84.6	13.5	9.6	29.8	9.9
	Mangochi Total				935	328		27.3	76.7	11.9	11.6	26.2	1.4
	Total				2918	1118		24.7	61.5	11.6	6.2	20.8	5.3

1 The exact location of Kapala was not recorded. ^2^ Transmission regions are defined as low intensity seasonal transmission (LIST); high intensity seasonal transmission (HIST) and perennial transmission (PT). ^3^ Microscopy prevalence is for any *Plasmodium* species. ^4^ PCR prevalence is for any species (*P. falciparum*, *P. malariae* or *P. ovale*) detected. ^5^ Fever is defined as axillary temperature ≥37.5°C. ^6^ Moderate anaemia is defined as Hb ≤8 g/dl. ^7^ People treated were at risk of progression to severe malaria, as defined in materials and methods. ^8^ People who slept regularly under a bednet.

### Age- and geographical-related variance in prevalence of Plasmodium species

Three out of four human malaria species (*P. falciparum*, *P. malariae* and *P. ovale*) were detected by PCR in both districts (Table S1). *P. vivax* was not detected in either district. The species diagnostic results from replicate microscopy readings were not reliable and therefore only PCR speciation was used. Microscopy density readings were therefore considered as total *Plasmodium* density. As with most other malaria endemic regions, *P. falciparum* predominated in both districts (Dedza, 53.2%; Mangochi 75.7%), whilst *P. malariae* (Dedza, 7.7%; Mangochi 13.2%) and *P. ovale* (Dedza, 3.3%; Mangochi 7.8%) were observed less frequently and usually only in combination with *P. falciparum* (Table S1).

**Figure 4:**
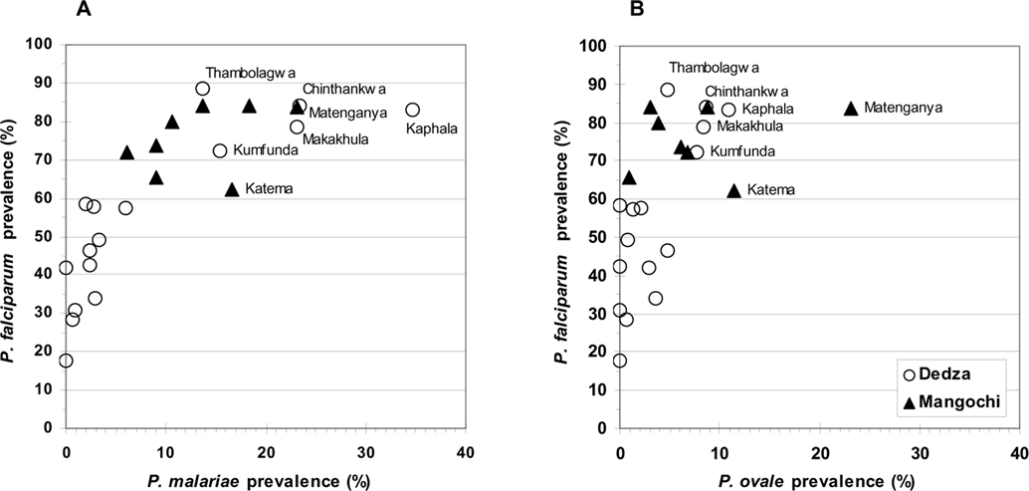
Plots of prevalence of *P. falciparum* against *P. malariae* (A) and *P. falciparum* against *P. ovale* prevalence (B) for individual villages in Dedza and Mangochi districts. Names are shown for the 5 Dedza villages in the HIST region of Dedza and 1 Mangochi village (Matenganya) with the highest *P. ovale* prevalence, for ease of comparison.

**Figure 5:**
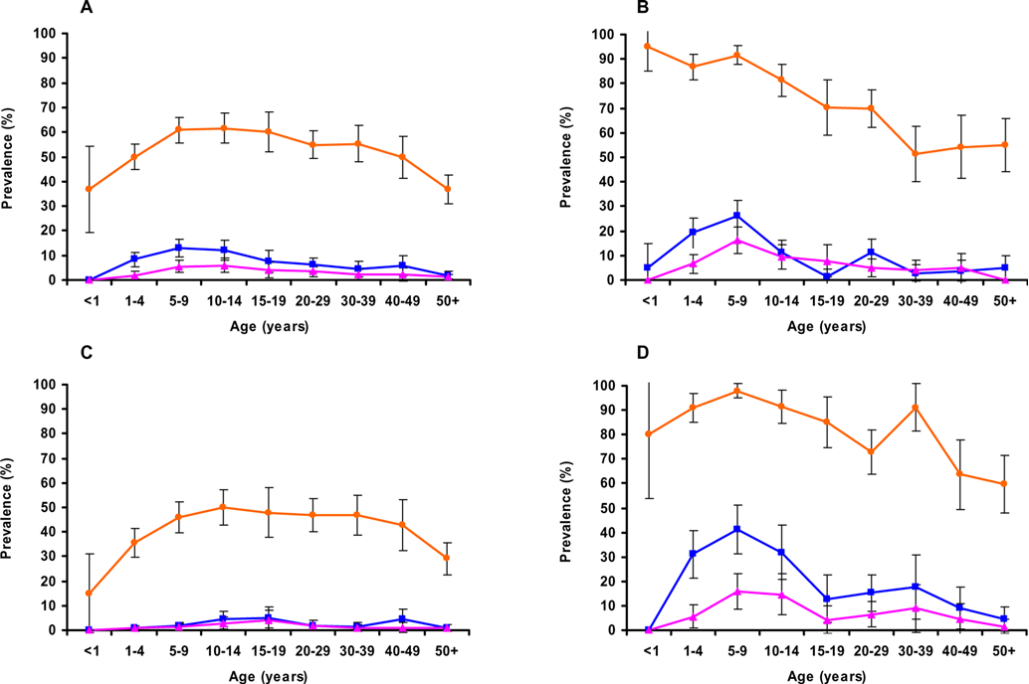
*Plasmodium* species-specific age-prevalence profiles determined by PCR. Total populations of Dedza district (A), Mangochi district (B), and for Dedza district sub-divided by transmission region, LIST (C) and HIST (D). Data for each *Plasmodium* species are as follows: *P. falciparum* (orange), *P. malariae* (blue) and *P. ovale* (pink). Error bars show 95% confidence intervals.

For all species, prevalence was greatest in Mangochi. As seen with total *Plasmodium* prevalence, the relative abundance of the three *Plasmodium* species showed striking variation between villages in Dedza but was less variable in Mangochi (Figure 4). The five Dedza villages in the high intensity transmission region had *P. falciparum* prevalence values within the range of those for Mangochi villages, and had a correspondingly high prevalence of *P. malariae* (Figure 4a) and *P. ovale* infections (Figure 4b). One Mangochi village (Matenganya) showed more than double the *P. ovale* prevalence of the other Mangochi villages (Figure 4b). The age-prevalence profiles of each of the *Plasmodium* species show marked differences between the two districts (Figure 5a and b). *P. falciparum* prevalence in Dedza increased with age from approximately 35–60% in the years 1–4, thereafter maintaining a stable level of between 50 and 60% (Figure 5a). In contrast, *P. falciparum* prevalence in Mangochi was around 90% in infants and children and declined with age during adolescence, reaching similar levels amongst adults to those found in Dedza. *P. malariae* and *P. ovale* age profiles are similar in Dedza, showing a slight but not significant rise in children aged 5–14 years. In Mangochi, the two species also gave similar patterns but with a pronounced peak amongst children aged 5–9 years (Figure 5b). When the Dedza data were separated into LIST and HIST regions, significant differences were observed between the regions in the age-prevalence pattern, with the profile for the LIST region showing a peak-shift towards older individuals for *P. malariae* and *P. ovale* infection and the HIST region profile closely resembling that of Mangochi (Figure 5c and d).

### Distribution of multiple species infections

To determine if the three *Plasmodium* species were randomly distributed within human hosts we used the multiple-kind lottery model [29] which detects deviations from a random distribution of multiple parasite species within multiple hosts. Because of the significant age and spatial differences in prevalence, data from children and adults were analysed separately within each of the three transmission regions. No deviation from a random distribution was seen in children from any transmission region when all species (*P. falciparum*, *P. malariae* and *P. ovale*) were considered or when *P. falciparum* and *P. malariae* were considered in isolation from the least prevalent species, *P. ovale* (Table 4). A greater number of mixed infections in the LIST region and fewer *P. malariae* infections in Mangochi were observed amongst adults compared with that expected from a random distribution of each species (Table 4). Combining data from children and adults led to the abolition of randomness in all of the regions where randomness had been found in children, indicating that differences in the number and distribution of infections between age groups can confound analyses of whole populations. In addition, combining data for the two Dedza transmission regions also resulted in apparent deviation from randomness amongst children.

**Table 4:** Analysis of the distribution of mixed-species infections within children and adults from the 3 transmission regions.

Transmission Region	Children only		Adults only		All ages combined	
	All species	Pf and Pm only	All species	Pf and Pm only	All species	Pf and Pm only
Dedza, LIST	Random distribution	Random distribution	Excess mixed infections	Excess mixed infections	Excess mixed infections	Excess mixed infections
	R_OE Mixed_ = 1.38 (0.66–2.09)	R_OE Mixed_ = 1.52 (0.53–2.51)	R_OE Mixed_ = 1.73 (1.00–2.46)	R_OE Mixed_ = 1.97 (0.95–2.99)	R_OE Mixed_ = 1.57 (1.05–2.08)	R_OE Mixed_ = 2.61 (1.55–3.66)
	p = 0.40	p = 0.41	**p = 0.019**	**p = 0.008**	**p = 0.012**	**p≤0.0001**
Dedza, HIST	Random distribution	Random distribution	Random distribution	Random distribution	Excess negatives	Fewer Pm infections
	R_OE Mixed_ = 0.99 (0.84–1.14)	R_OE Mixed_ = 1.05 (0.87–1.23)	R_OE Mixed_ = 1.08 (0.75–1.14)	R_OE Mixed_ = 1.13 (0.72–1.53)	R_OE Negative_ = 1.27 (1.02–1.51)	R_OE Pm_ = 0.37 (0.12–0.63)
	p = 0.23	p = 0.22	p = 0.60	p = 0.66	**p = 0.030**	**p = 0.004**
Dedza regions combined	Excess mixed infections	Excess mixed infections	Excess mixed infections	Excess mixed infections	Excess mixed infections	Excess mixed infections
	R_OE Mixed_ = 1.44 (1.19–1.69)	R_OE Mixed_ = 1.66 (1.34–1.97)	R_OE Mixed_ = 1.49 (1.11–1.87)	R_OE Mixed_ = 1.63 (1.14–2.12)	R_OE Mixed_ = 1.48 (1.27–1.69)	R_OE Mixed_ = 1.68 (1.41–1.95)
	**p = <0.0001**	**p = <0.0001**	**p = 0.0009**	**p = 0.0001**	**p≤0.0001**	**p≤0.0001**
Mangochi, PT	Random distribution	Random distribution	Excess mixed infections	Fewer Pm infections	Excess negatives	Fewer Pm infections
	R_OE Mixed_ = 0.98 (0.83–1.14)	R_OE Mixed_ = 1.08 (0.89–1.28)	R_OE Mixed_ = 1.45 (1.01–1.89)	R_OE Pm_ = 1.43 (0.85–2.01)	R_OE Negative_ = 1.20 (1.06–1.34)	R_OE Pm_ = 0.27 (0.08–0.45)
	p = 0.14	p = 0.11	**p = 0.013**	**p = 0.048**	**p = 0.0007**	**p≤0.0001**

Two types of analysis were carried out using the Multiple-kind Lottery Model [29]: all *Plasmodium* species (*P. falciparum*, *P. malariae* and *P. ovale*) and *P. falciparum* and *P. malariae* only. R_OE Mixed_, R_OE Negative_ or R_OE Pm_ (95% CI) is the ratio of observed/expected infections that deviated from expected values and is a measure of the magnitude of deviation from randomness. P values less than 0.05 are in bold.

### Parasite Density

**Figure 6:**
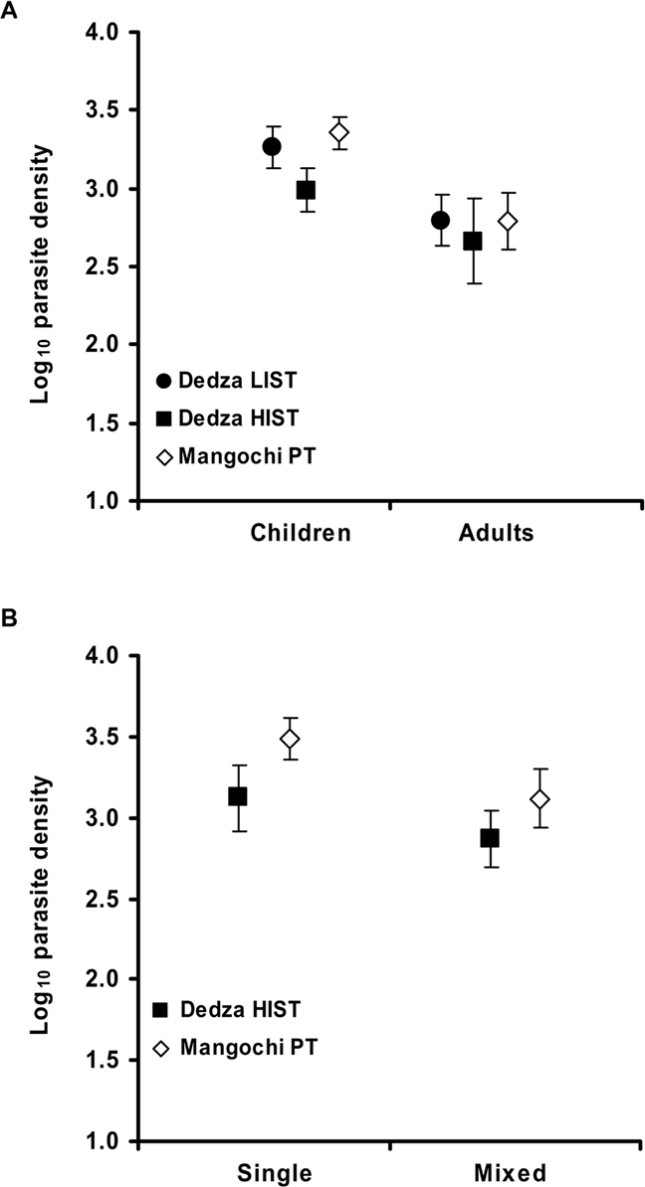
Mean log_10_ parasite density with 95% confidence intervals. (A) children and adults in the 3 transmission regions and (B) children with single and mixed species infections from HIST (Dedza) and PT (Mangochi) transmission regions.

Generally, parasite density is closely linked with malaria fever, with higher densities more likely to result in elevated temperature. Parasite density measured by microscopy was greater in Mangochi (mean log_10_ density per µl of blood, 3.2) than in Dedza, (mean log_10_ density, 3.0, Table 1). When estimates of the density of sub-microscopic parasites, detected only by PCR were included, this difference was abolished (Table 1). In all 3 transmission regions mean log_10_ parasite density was greater in children compared with adults (LIST region, p<0.0001; HIST region, p = 0.027; Mangochi PT region, p<0.0001, Figure 6a). Amongst adults there was no difference in parasite density between transmission regions but in children, density was lower in the HIST region compared with both the LIST (p = 0.0060) and the Mangochi PT regions (p<0.0001, Figure 6a). Children with mixed species infections detected by PCR, had lower mean parasite density than those with single infections in the PT region (p = 0.0021) but not in the HIST region (p = 0.071, Figure 6b). Children from the LIST region and adults from all regions showed no difference in parasite density between single or mixed infections but in all these comparisons the number of individuals with mixed infections was less than 10, limiting the strength of these comparisons.

### Fever

We assessed fever as the principal clinical feature of malaria, by measuring temperature at the time of blood sample collection and we also estimated the occurrence of fever during the previous 2 weeks using a questionnaire. In order to prevent bias in comparisons across regions and between single and mixed infections, we chose not to define regional- and age-specific clinical thresholds of fever and parasite density [30], [31], instead comparing fever alone and fever along with standard density thresholds.

Despite the differences in *Plasmodium* prevalence and species distribution between districts, the proportion of individuals with measured fever was very similar (Dedza, 11.5%, Mangochi, 11.9%, Table 1). Likewise, there was no difference in fever rates between the HIST and LIST transmission regions (LIST, 11.6%; HIST, 11.1%, p = 0.770). In both districts fever rates were higher in children than in adults (Dedza: children, 14.0%; adults 9.1%, OR 1.64 (95% CI 1.23–2.17) p = 0.0005); Mangochi: children 14.3%, adults 9.0%, OR 1.69 (95% CI 1.11–2.56), p = 0.013). However, there was no difference between districts or transmission regions in the child or adult fever rates (data not shown).

Fever was seen in 209 out of 1426 (14.6%) people with *P. falciparum* only, compared with 30 out of 203 (14.8%) people with mixed *P. falciparum* & *P. malariae* infections and 8 out of 79 (10.1%) people with mixed *P. falciparum* and *P. ovale* infections. These data showed no deviation from expected fever rates in either district when data from all age groups or children (aged <1–14 years) only were analysed (data not shown). There were insufficient data from each of the two Dedza transmission regions for these to be analysed separately. No significant deviations from expected fever rates amongst *P. falciparum* only and mixed-species infections were seen when clinical malaria was defined more stringently as temperature ≥37.5°C or 38.0°C along with a positive microscopy reading or along with parasite density ≥1000 parasites/µl (data not shown). Only when clinical malaria was defined more stringently as temperature ≥37.5°C along with parasite density ≥5000 parasites/µl, was there a negative association of mixed infections with clinical malaria, and this was seen in children only (OR 0.43 (95% CI 0.19–0.96) p = 0.035).

Univariate logistic regression was used to investigate the variables associated with measured fever. The variables *district* and *transmission region* were not significantly associated but 14 out of 25 other variables were (Table S2). Multivariate logistic regression modelling revealed that the best predictor of fever was log_10_ parasite density in the absence of all other factors but this model accounted only for 8.2% of the variability in fever (Table 5).

**Table 5:** Multivariate logistic regression modelling on fever (≥37.5°C), history of fever and moderate anaemia (Hb≤8.0 g/dl).

Model	Variables included in model	Odds Ratio (OR)	p-value
Fever	Log_10_ parasite density	0.67	<0.0001
	Nagelkerke R^2^ = 0.082		
History of fever in previous 2 weeks	Transmission region (overall)	-	**0.014**
	Transmission region LIST	Reference OR = 1	
	Transmission region HIST	0.003	0.99
	Transmission region PT	1.51	**0.011**
	Village (overall)	-	**0.0023**
	Age Group (overall)	-	**0.0004**
	<1 year	−0.73	0.30
	1–4 years	−1.09	**0.026**
	5–9 years	−1.46	**0.0027**
	10–14 years	−1.76	**0.0006**
	15–19 years	−1.75	**0.0025**
	20–29 years	−0.6	0.25
	30–39 years	−1.2	**0.033**
	40–49 years	−0.056	0.93
	50+ years	Reference OR = 1	
	Mild anaemia ([Hb]≤11 g/dl)	−0.88	**<0.0001**
	Log_10_ parasite density*	0.16	0.14
	Sleeping regularly under a bednet	−1.22	**0.0082**
	Nagelkerke R^2^ = 0.230		
Anaemia	Transmission region overall	-	**0.012**
	Transmission region LIST	Reference OR = 1	
	Transmission region HIST	−0.816	**0.0041**
	Transmission region PT	−0.562	0.081
	Age group (overall)	-	**<0.0001**
	<1 year	1.5	**0.043**
	1–4 years	0.23	0.68
	5–9 years	−2.08	**0.0011**
	10–14 years	−2.77	**0.0017**
	15–19 years	−1.57	0.078
	20–29 years	−1.57	**0.031**
	30–39 years	−1.19	0.132
	40–49 years	−1.91	0.095
	50+ years	Reference OR = 1	
	Log_10_ parasite density	0.68	**<0.0001**
	Nagelkerke R^2^ = 0.330		

* The inclusion of this variable substantially increased the fit of the model despite the p-value not being significant. P values less than 0.05 are in bold.

A history of fever during the 2 weeks previous to the community survey was self-reported by more than 40% of people in both districts (Dedza, 45.8%, Mangochi, 42.5%, Table 1). The HIST region showed a slightly greater rate of history of fever (50.6%) than both the LIST (43.9%) or PT regions (42.5%) (HIST cf LIST, OR 1.31 (95% CI 1.07–1.60), p = 0.0076; HIST cf PT, OR 1.39 (95% CI 1.12–1.72), p = 0.0028). The LIST region showed a higher rate of history of fever in adults (47.5%) compared with children (40.2%), (OR 1.34 (95% CI 1.09–1.66), p = 0.0057) whereas no difference between age groups was seen in the other transmission regions (data not shown). People reporting a history of fever were less likely to have mixed species infection than expected by chance in the HIST region (OR 0.63 (95% CI 0.42–0.94), p = 0.025) but this was not the case in the LIST or Mangochi regions (data not shown). Almost one quarter of the variability in the history of fever (23.0%) could be predicted using multivariate logistic regression modelling (Table 5). This is in contrast to measured fever, which was poorly predicted by such analysis.

### Antimalarial treatment, drug and bednet use

During the community surveys, we gave antimalarial treatment (see Materials and Methods for clinical treatment criteria) to a greater proportion of people in Mangochi (26.2%) than in Dedza (18.3%, Table 1). Children were more likely than adults to meet treatment criteria in both districts (Dedza: children 24.0%, adults 13.0%; Mangochi: children 33.6%, adults 17.3%) and both children and adults in Mangochi were more likely to require treatment than equivalent individuals in Dedza (children, OR 1.60 (95% CI 1.27–2.03), p = <0.0001; adults, OR 1.40 (95% CI 1.03–1.91), p = 0.033). There was no association between meeting treatment criteria and carriage of multiple species infection; individuals who were treated had no more or less mixed species infections than expected by chance alone when data were analysed by district, transmission region or age (data not shown).

Drug use by each participant in the two weeks previous to the community survey was estimated via a questionnaire. There was no difference between districts in the proportion of people that had taken the anti-malarial drugs SP or quinine (Dedza 7.4%, Mangochi 8.9%) but more people in Mangochi had taken painkillers (Dedza 21.6%, Mangochi 26.7%, Table 1). In both Dedza and Mangochi districts, people who had taken antimalarial treatment in the previous 2 weeks were less likely to have a mixed species infection (Dedza, OR 0.10 (95% CI 0.02–0.40), p<0.0001; Mangochi, OR 0.31 (95% CI 0.11–0.89), p = 0.022).

Bednet usage varied between the two districts. Only 1.4% of individuals in Dedza slept under a bednet regularly, compared with 14.2% in Mangochi (Table 1). In Mangochi, people who slept regularly under a bednet were less likely to have a *Plasmodium* infection detected by PCR (OR 0.43, 95% CI 0.29–0.65, p<0.0001) and had fewer mixed species infections than expected by chance alone (OR 0.22, 95% CI 0.09–0.55, p = 0.0004). There was a near-significant association between bednet use and reduced current fever rate (OR 0.52, 95% CI 0.26–1.07, p = 0.07) but surprisingly, those using bednets were more likely to have a history of fever than those without (OR 1.82, 95% CI 1.24–2.69, p = 0.002). The low numbers of bednet users prevented similar analyses for Dedza district.

### Haemoglobin concentration and anaemia

**Figure 7:**
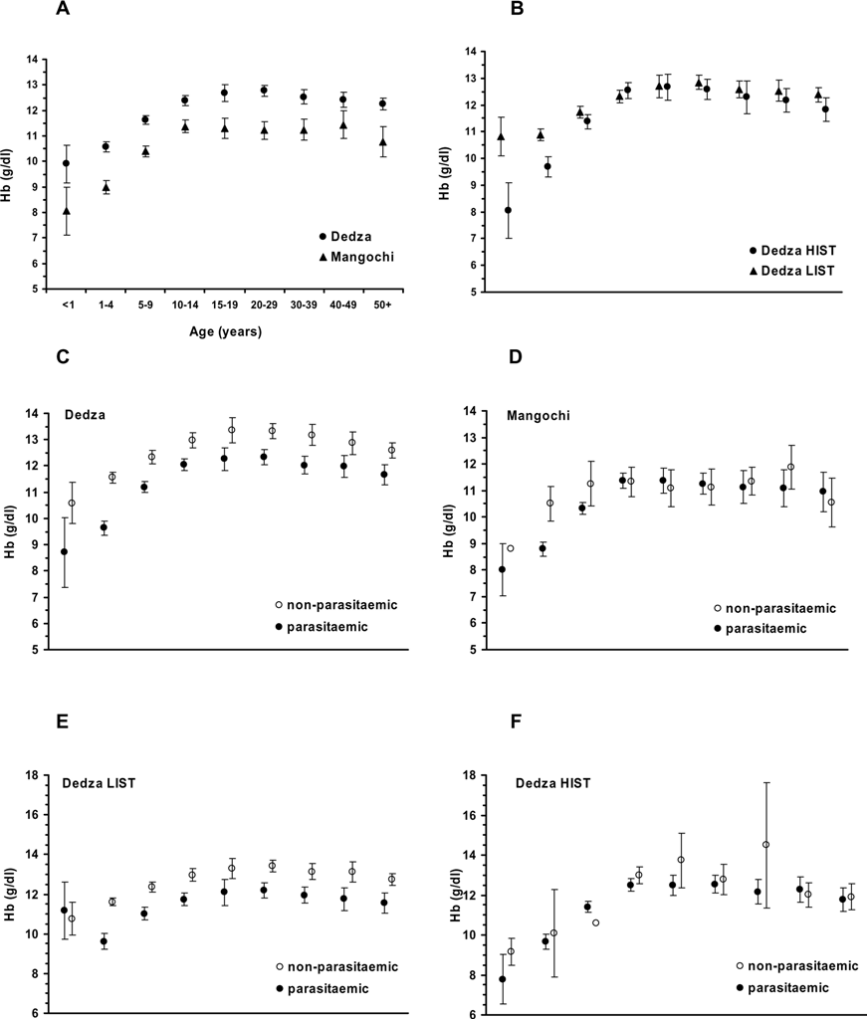
Effect on age-specific mean haemoglobin concentration ([Hb]) of district (A), transmission region (B) and parasite status detected by PCR (C–F). Error bars show 95% confidence intervals. Where error bars are absent, the datum point represents a single individual. Note different y-axis scales on E and F.

Overall, mean haemoglobin concentration ([Hb]) in Mangochi (10.6 g/dl) was significantly lower than Dedza (12.0 g/dl, Table 1). Correspondingly, levels of anaemia were different in the two districts. Mild anaemia ([Hb]≤11 g/dl) affected 55% of people in Mangochi, but was only half as prevalent in Dedza. Levels of moderate anaemia ([Hb]≤8 g/dl) in Mangochi were three times higher than in Dedza (Table 1). Mean haemoglobin concentration increased with age in both districts, stabilising in adulthood but was consistently lower (by approximately 1.5 g/dl) across all age groups in Mangochi (Figure 7a). When the two Dedza transmission regions were considered separately, a higher mean [Hb] was found in the LIST region compared with the HIST region in children less than 5 years old, but in older children and adults there was no difference (Figure 7b).

Mean haemoglobin concentration was lower in parasitaemic individuals than in those without parasitaemia across all age groups in Dedza (Figure 7c). When analysed separately, the LIST region mean [Hb] was reduced by around 1 g/dl in parasitaemic individuals across ages except for the youngest age group (children <1 year, Figure 7e). In contrast, mean [Hb] in parasitaemic people of all ages was indistinguishable from those without parasitaemia in the HIST region (Figure 7f). In Mangochi, there was no difference in mean [Hb] between parasitaemic and non-parasitaemic individuals, except for children aged 1–4 in whom mean [Hb] was around 2 g/dl lower amongst parasitised children (Figure 7d). There was no difference in mean [Hb] in adults with single compared with mixed species infections in all transmission regions and in children in the LIST and PT transmission regions. However, in the HIST region, children with mixed species infections had greater mean [Hb] compared with those carrying a single species (single, 10.7 g/dl, n = 147, mixed 11.4 g/dl, n = 101, p<0.01).

Adults sleeping regularly under a bednet had higher mean [Hb] than those without bednets in Mangochi (mean [Hb] without net, 11.0 g/dl; with net, 11.7 g/dl, p = 0.018). In children, mean [Hb] did not differ with bednet use (mean [Hb] without net, 10.1 g/dl; with net, 10.2 g/dl, p = 0.540). The impact of bednet usage on [Hb] could not be analysed within Dedza district because so few people in this district used bednets (n = 28).

In order to determine associations between haemoglobin concentration and any of the variables that we measured, regression analyses were carried out. In a univariate regression analysis of [Hb], *transmission region* was strongly associated (R^2^ = 0.094, p<0.0001) therefore we carried out bi-variate regression analyses accounting for *transmission region*. All but 3 of the 24 measured variables showed significant association with haemoglobin level. There were significant interactions between *transmission region* and several variables (Table S3). Modelling of [Hb] using multivariate linear regression resulted in a best-fit model that explained 37.2% of the variability in [Hb] (Table 6).

**Table 6:** Multivariate linear regression modelling of haemoglobin concentration.

Model	Variables included in model	F statistic	p-value
[Hb]	Transmission region	6.13	**0.0023**
	Age group	2.34	**0.017**
	Fever (≥37.5°C)	8.21	**0.0043**
	Log_10_ parasite density*	3.77	0.052
	R^2^ = 0.372		

* The inclusion of this variable substantially increased the fit of the model despite the p-value not being significant. P values less than 0.05 are in bold.

*Transmission region* was also strongly associated with the binary clinical outcome of moderate anaemia ([Hb]≤8.0 g/dl) in univariate logistic regression (p<0.0001) and therefore we carried out bi-variate logistic regression analysis accounting for *transmission region*. When *transmission region* was included, 15 out of 24 measured variables were associated with anaemia (Table S3). Modelling of moderate anaemia using multivariate logistic regression resulted in a best-fit model that accounted for one third of variability in anaemia outcome (Table 5).

## Discussion

In this study we have investigated the impact of infection with multiple species of *Plasmodium*, on the clinical manifestations of malaria in different epidemiological settings. We described the epidemiology of multiple *Plasmodium* infections in two districts of Malawi with seasonal (Dedza) and perennial (Mangochi) malaria transmission. Within the seasonal transmission zone, we have explored the relationship of malaria prevalence with geophysical features and defined two regions in which prevalence varies markedly, almost certainly arising from elevation-linked variation in transmission intensity.

There were marked differences in the spatial and age-distribution of the three sympatric *Plasmodium* species detected (*P. falciparum*, *P. malariae* and *P. ovale*) between the three transmission regions. Prevalences of parasitaemia for each species were higher and peaked at a younger age in areas with high intensity seasonal or perennial transmission. In Dedza, high parasite prevalences were associated with low altitude and proximity to marshy ground. Higher *P. falciparum* prevalence was generally reflected in higher prevalence of minority species (*P. malariae* and *P. ovale*) raising the possibility of multiple *Plasmodium* species being co-transmitted. The relative abundance of these species showed micro-geographic variation. This could result from variation in the presence of mosquito species or sub-species, which may have *Plasmodium* species-specific transmission efficiencies. Alternatively, these differences could arise from stochastic variation in the seeding of infections by incoming human carriers within villages. The human populations in our two study areas come from different ethnic groups, the Chewa and Yao. Different ethnicity or religion can affect housing type, sleeping arrangements and bednet use – factors which may impact on mosquito exposure. However, we know of no evidence to suggest that these groups differ in their genetic predisposition or ability to raise immune responses to malaria infections.

This study is the first to describe the prevalence of *P. ovale* in Malawi, although its presence has been noted previously [32]. *P. vivax* was not detected in our study populations, despite its presence in neighbouring Mozambique [33] and the potential for importation via Malawi's trade links [34]. The distribution of the three *Plasmodium* species amongst the human population showed randomness amongst children in all regions but was not random in adults from the PT and LIST regions. A non-random distribution is thought to result from the effects of acquired immunity. Our data contrast with those from Papua New Guinea, a holoendemic region where all four co-endemic human malaria species show random distribution across all age groups [35].

Fever rates were remarkably similar between the three transmission regions despite differences in the seasonality and intensity of transmission and the age-prevalence profiles of each *Plasmodium* species. The lack of difference could not be explained by differences in anti-malarial drug use, which was similar between districts. People with a history of fever were more likely to have a single infection than those without but they were also more likely to have taken anti-malarial drugs. In 1998, SP (the national first line anti-malarial during study) had a 2-week efficacy of 80% in symptomatic *P. falciparum* cases in Blantyre (170 km from our study sites) [36]. However, 4-week parasitological failure rates were ∼30–60%, which may account for our observation that many individuals were parasitaemic despite recent self-treatment. In both Dedza and Mangochi, people who had taken antimalarial treatment in the previous 2 weeks were less likely to have a mixed species infection, suggesting that treatment was effective at least against *P. malariae* and *P. ovale* co-infections, if not always against *P. falciparum*. *In vivo* drug resistance of *P. malariae* has been described only for chloroquine [37] and genetic and *in vitro* studies failed to find evidence of anti-folate resistance in this species in Africa [38]. Differences in drug sensitivity resulting from variation in drug selection pressure and/or mutability between *Plasmodium* species could explain these data.

Fever was equally likely in people with single or mixed infections. Only when clinical malaria was defined as fever ≥37.5°C with parasite density ≥5000 parasites/µl were there fewer mixed infections than expected, amongst children only. Parasite density was lower amongst children with mixed infections than those with single-species infections, but only in the PT region. This phenomenon may result from a greater level of acquired immunity under perennial transmission, resulting from the earlier age at infection and greater number of infections, acting to reduce density in more heavily infected children. Changes to the immunological control of parasite density and tolerance under different transmission settings can account for differences in *P. falciparum* morbidity [39] and similar mechanisms for each species may reduce all species densities. A reduction in parasite density in children with mixed-species infections described in Tanzania [5] was accompanied by a reduction in fevers, but we found no reduction in fevers in our study. It is possible that lowering of the fever rate (resulting from a reduction in parasite density) in Mangochi was negated by the effect of greater or sustained transmission. The absence of a protective effect of multiple infection against fever, except amongst children with the highest parasite densities, is also in contrast to previous findings from Cote d'Ivoire where children with fevers had fewer mixed *P. falciparum*-*P. malariae* infections than asymptomatic children [4].

A possible explanation for these differences is that in children with very high *P. falciparum* densities, the detection of lower density species by PCR is masked. This phenomenon is known to occur when detecting multiple *P. falciparum* genotypes using common primers [40]. The nested species-specific PCR of Snounou *et al.*,which we employed in this study, also uses common primers in the first round of amplification. This problem could be overcome by designing new PCR methods for malaria diagnosis in which each species was detected independently, without any amplification primers in common. This “common primer” explanation is given weight by the results of our analysis using different density thresholds and the less compelling results given in the Cote d'Ivoire study for children selected on less stringent clinical criteria, who may have had lower parasite densities. Differences in case-definitions of clinical malaria may underlie a similar discrepancy with data from Papua New Guinea [8].

Anaemia has long been recognised as a complication of malaria, but only recently have studies have confirmed the causal link at the community level [41], [42]. Lowered [Hb] in malaria infection is due to the destruction of infected red blood cells during asexual multiplication, immune-mediated destruction of non-infected red blood cells [43] and dyserythropoiesis [44]. In our study, people living under perennial malaria transmission (Mangochi) had a lower mean [Hb] across all age groups than those in the seasonal transmission district (Dedza), and had a greater incidence of anaemia. The lowered [Hb] in Mangochi is probably due to perpetual malaria challenge, although from this study we cannot exclude alternative and unrelated explanations related to the multifactorial etiology of anaemia. Previous studies have determined the effect of micro-nutrient deficiencies, haemoglobinopathies and G6PD variants in Malawi [45]. Cultural differences between the Chewa and Yao can affect height-for-age in children under 5 years in a complex interaction with other factors and there are differences in the levels of growth stunting in these two ethnic groups, suggestive of differences in nutrition [46]. HIV and helminth infections are also known to be important factors in anaemia. There is little difference in the prevalence of soil transmitted helminths between the two study districts but there are higher levels of schistosomiasis in lakeside compared with highland areas [47]. There is a large difference in the prevalence of HIV, which is twice as high in Mangochi as in Dedza, according to official statistics [24].

An association of anaemia with perennial malaria transmission [48] is consonant with a greater proportion of severe anaemia and greater rate of blood transfusion amongst paediatric admissions in Mangochi compared with Dedza (unpublished observations). It is likely that persistent malaria pressure resulting in lowered [Hb] puts children at risk of life-threatening anaemia, especially when compounded with other factors such as micronutrient deficiency. The presence of malaria parasites was a useful indicator of [Hb] at the individual level in the LIST region but not in the HIST or PT regions. The apparent lack of effect of parasitaemia on [Hb] in the higher transmission regions (other than in young children) is likely to be because detection of parasites in cross-sectional sampling is a poor predictor of parasitaemia status in older children and adults, in whom low density, chronic infections are not always detectable [49]–[51].

The higher parasite density found in children compared to adults in all transmission regions may account for the lower mean [Hb] in children relative to adults. Children with mixed species infections had on average greater [Hb] than children with single infections, suggestive of a protective effect of multiple infections. However, this finding was only seen in the HIST region. The functional mechanism for this may be specific to immunity generated through seasonal, compared to perennial infection. The counter-intuitive, lower parasite density found in mixed species infections could result from the inherent bias in the PCR species diagnosis, in which mixed infections may be more likely to be detected in patients where the density of each species is equitable. *P. malariae* and *P. ovale* produce lower density parasitaemia than *P. falciparum* and therefore mixed infections with *P. falciparum* may be more likely to be detected at lower overall densities.

The use of insecticide treated bednets is now a central focus of anti-malarial campaigns [52]. Bednet use at the time of our study in 2002 was very low although this increased considerably in subsequent years [53]. Bednet users in Mangochi had fewer mixed species infections than those not using nets, in keeping with previous data indicating the role of bednets in preventing new infections [54]. Use of a bednet was associated with a higher mean [Hb] in adults but surprisingly not in children in Mangochi, despite this effect having been described in children in other studies in Malawi [55] and other African countries [56]. Bednet use gave no protection against fever and those with bednets were more likely to have a history of fever than those without, in contradiction to the multiple studies showing the clinical benefit of bednets against malaria. These results may be due to poor power in this study resulting from the limited number of bednet users.

This study took place during the peak of malaria transmission in the wet season. Dry season data would allow a greater understanding of the differences between the perennial and seasonal transmission regions. It would be useful to clarify the relationship between the HIST and LIST regions to establish the dynamics of carriage of each species during the dry season in these populations. An understanding of how the prevalence of each *Plasmodium* species decays during the dry season, and the corresponding effect on [Hb] and fever would give valuable insights into the dynamics of the relationships between these factors. Our study suggests that the interaction of multiple *Plasmodium* species can have protective effects against some clinical outcomes of malaria. This protection appears to be highly dependent on the level and seasonality of malaria transmission and most likely results from differences in the dynamics of acquired immunity. Our data will be informative in predicting the effects of anti-malarial intervention strategies on the clinical outcomes of malaria and on *Plasmodium* population dynamics across multiple transmission settings.

## Supporting Information

Table S1 The number of samples in which each combination of Plasmodium species was detected by PCR for each of the 3 transmission regions.
(0.05 MB DOC)

Table S2 Univariate logistic regression analysis on fever (axillary temperature ≥37.5°C).
(0.07 MB DOC)

Table S3 Bi-variate linear regression analysis on haemoglobin concentration and bi-variate logistic regression on moderate anaemia ([Hb]≤8.0 g/dl), accounting for transmission region.
(0.09 MB DOC)
