# Supplementary material for: Effect of Transmission Setting and Mixed Species Infections on Clinical Measures of Malaria in Malawi
Source: PLoS One. 2008 Jul 23;3(7):e2775. doi: 10.1371/journal.pone.0002775 (PMC2467490; doi:10.1371/journal.pone.0002775)
Supplement: Table S1 — The number of samples in which each combination of Plasmodium species was detected by PCR for each of the 3 transmission regions. (0.05 MB DOC) [file pone.0002775.s001.doc]

**Table S1**

|  | Dedza, LIST | Dedza, HIST | Mangochi, PT | Total |
| --- | --- | --- | --- | --- |
| Negative for all *Plasmodium* species | 818 | 87 | 218 | 1123 |
| *P. falciparum* only | 568 | 316 | 548 | 1432 |
| *P. malariae* only | 8 | 7 | 6 | 21 |
| *P. ovale* only | 6 | 1 | 1 | 8 |
| *P. falciparum* & *P. malariae* | 19 | 94 | 90 | 203 |
| *P. falciparum* & *P. ovale* | 12 | 22 | 45 | 79 |
| *P. malariae* & *P. ovale* | 0 | 1 | 2 | 3 |
| *P. falciparum*, *P. malariae* & *P. ovale* | 4 | 20 | 25 | 49 |
|  |  |  |  |  |
| Total *P. falciparum* positive | 603 | 452 | 708 | 1763 |
| Total *P. malariae* positive | 21 | 122 | 123 | 276 |
| Total *P. ovale* positive | 32 | 44 | 73 | 139 |
|  |  |  |  |  |
| Total number of samples | 1435 | 548 | 935 | 2918 |
