# Supplementary material for: Effect of Transmission Setting and Mixed Species Infections on Clinical Measures of Malaria in Malawi
Source: PLoS One. 2008 Jul 23;3(7):e2775. doi: 10.1371/journal.pone.0002775 (PMC2467490; doi:10.1371/journal.pone.0002775)
Supplement: Table S2 — Univariate logistic regression analysis on fever (axillary temperature ≥37.5°C). (0.07 MB DOC) [file pone.0002775.s002.doc]

**Table S2**

#

| Variable | Odds Ratio (OR) | p-value | p-value for interaction with district | p-value for interaction with transmission region |
| --- | --- | --- | --- | --- |
| District | -0.045 | 0.72 | - | - |
| Transmission region | - | 0.90 | - | - |
| Transmission region HIST | -0.032 | 0.80 | - | - |
| Transmission region PT | -0.079 | 0.64 | - | - |
| Transmission region LIST | Ref OR=1 | - | - | - |
| Village | - | **0.0015** | - | 0.053 |
| Sex | -0.096 | 0.42 | 0.33 | 0.44 |
| Age (years) | -0.017 | **<0.0001** | 0.78 | **0.0010** |
| Age group (overall) | - | **0.0015** | 0.050 | 0.35 |
| Age group <1 year | 0.965 | **0.0291** | - | - |
| Age group 1-4 years | 0.938 | **0.0002** | - | - |
| Age group 5-9 years | 0.804 | **0.0013** | - | - |
| Age group 10-14 years | 0.638 | **0.0170** | - | - |
| Age group 15-19 years | 0.581 | 0.058 | - | - |
| Age group 20-29 years | 0.434 | 0.11 | - | - |
| Age group 30-39 years | 0.439 | 0.14 | - | - |
| Age group 40-49 years | -0.010 | 0.98 | - | - |
| Age group 50+ | Ref OR=1 | - | - | - |
| Adult/child grouping | 0.503 | **<0.0001** | 0.87 | 0.97 |
| Christian religion | 0.031 | 0.80 | 0.55 | 0.84 |
| Haemoglobin concentration (g/dl) | -0.208 | **<0.0001** | **0.045** | 0.11 |
| Anaemia ([Hb] ≤11.0 g/dl) | -0.799 | **<0.0001** | 0.31 | 0.49 |
| Anaemia ([Hb] ≤8.0 g/dl) | -0.703 | **0.0003** | 0.60 | 0.85 |
| Malaria positive by microscopy | -1.06 | **<0.0001** | 0.16 | 0.29 |
| Malaria positive by PCR | -0.748 | **<0.0001** | 0.21 | 0.54 |
| Log_10_ parasite density | 0.673 | **<0.0001** | **0.023** | **0.049** |
| Log_10_ parasite density including PCR | 0.478 | **<0.0001** | 0.056 | 0.055 |
| *P. falciparum* positive by PCR | -0.747 | **<0.0001** | 0.69 | 0.68 |
| *P. malariae* positive by PCR | -0.216 | 0.24 | 0.97 | 0.65 |
| *P. ovale* positive by PCR | 0.087 | 0.76 | 0.60 | 0.79 |
| Mixed species infection | 0.117 | 0.51 | 0.20 | **0.015** |
| Number of species | 0.349 | **<0.0001** | 0.19 | **0.009** |
| *P. falciparum* & *P. malariae* mixed infection | -0.300 | 0.15 | 0.86 | 0.75 |
| *P. falciparum* & *P. ovale* mixed infection | 0.158 | 0.68 | 0.83 | 0.83 |
| Treated during study | -6.85 | **<0.0001** | **<0.0001** | **<0.0001** |
| History of fever in previous 2 weeks | -0.292 | **0.012** | 0.13 | 0.31 |
| Taken anti-malarial in previous 2 weeks | -0.029 | 0.89 | 0.25 | 0.51 |
| Taken painkiller in previous 2 weeks | -0.032 | 0.82 | 0.49 | 0.32 |
| Sleeping regularly under a bednet | 0.347 | 0.24 | 0.17 | 0.39 |
| Spent night away in previous 4 weeks | 0.758 | 0.10 | 0.57 | 0.85 |
|  |  |  |  |  |

# * Odds ratios for categorical variables with more than 2 categories are not given. P values less than 0.05 are in bold
