# Supplementary material for: Effect of Transmission Setting and Mixed Species Infections on Clinical Measures of Malaria in Malawi
Source: PLoS One. 2008 Jul 23;3(7):e2775. doi: 10.1371/journal.pone.0002775 (PMC2467490; doi:10.1371/journal.pone.0002775)
Supplement: Table S3 — Bi-variate linear regression analysis on haemoglobin concentration and bi-variate logistic regression on moderate anaemia ([Hb]≤8.0 g/dl), accounting for transmission region. (0.09 MB DOC) [file pone.0002775.s003.doc]

**Table S3**

|  | Haemoglobin concentration | | | Moderate Anaemia | | |
| --- | --- | --- | --- | --- | --- | --- |
| Variable | R^2^ | Other variable  p-value | Interaction  p-value | Other  variable  Odds Ratio | Other  variable  p-value | Interaction  p-value |
| Village | 0.125 | **<0.0001** | - | - | 0.11 | **<0.0001** |
| Sex | 0.100 | **0.028** | 0.24 | -0.042 | 0.80 | 0.14 |
| Age (years) | 0.138 | **<0.0001** | 0.11 | -0.022 | **<0.0001** | 0.11 |
| Age group | 0.261 | **<0.0001** | **0.0006** | - | **<0.0001** | 0.73 |
| Adult/child grouping | 0.170 | **<0.0001** | 0.33 | 0.815 | **<0.0001** | 0.20 |
| Axillary temperature | 0.112 | **<0.0001** | **0.021** | 0.308 | **0.0036** | **<0.0001** |
| Fever (axillary temperature ≥37.5^o^C) | 0.120 | **<0.0001** | 0.33 | -0.715 | **<0.001** | **0.0029** |
| History of fever in previous 2 weeks | 0.124 | **<0.0001** | **0.0002** | -0.771 | **<0.0001** | 0.056 |
| Microscopy positive | 0.160 | **<0.0001** | **0.029** | -1.507 | **<0.0001** | **0.0040** |
| Log_10_ parasite density | 0.135 | **<0.0001** | 0.69 | 0.833 | **<0.0001** | 0.55 |
| Log_10_ parasite density including PCR | 0.125 | **<0.0001** | 0.060 | 0.624 | **<0.0001** | **0.0062** |
| Any species detected by PCR | 0.148 | **<0.0001** | **0.0017** | -1.21 | **<0.0001** | 0.098 |
| *P. falciparum* detected by PCR | 0.145 | **<0.0001** | **0.0042** | -1.02 | **<0.0001** | 0.11 |
| *P. malariae* detected by PCR | 0.107 | **<0.0001** | **0.013** | 0.107 | 0.66 | 0.51 |
| *P. ovale* detected by PCR | 0.101 | 0.068 | 0.12 | -0.553 | 0.17 | 0.59 |
| Number of species detected by PCR | 0.141 | **<0.0001** | **<0.0001** | 0.329 | **0.0035** | **0.0016** |
| Mixed infection detected by PCR | 0.100 | **0.0097** | 0.24 | 0.251 | 0.30 | 0.67 |
| *P. falciparum* and *P. malariae* mixed infection | 0.105 | **<0.0001** | 0.24 | 0.029 | 0.92 | 0.81 |
| *P. falciparum* and *P. ovale* mixed infection | 0.101 | **0.010** | 0.63 | 0.886 | 0.14 | 0.86 |
| Christian religion | 0.100 | 0.070 | 0.38 | 0.355 | 0.052 | 0.79 |
| Taken antimalarial in previous 2 weeks | 0.107 | **0.0002** | **0.0091** | -0.601 | **0.012** | 0.11 |
| Taken painkiller in previous 2 weeks | 0.114 | **<0.0001** | **0.0004** | -0.590 | **0.0005** | 0.76 |
| Sleeps regularly under bednet | 0.100 | 0.068 | 0.96 | 0.766 | **0.043** | 0.24 |
| Spent night away in previous 4 weeks | 0.105 | **<0.0001** | 0.54 | 1.67 | 0.096 | 0.72 |
|  |  |  |  |  |  |  |

# Interaction p-values denote interactions between *transmission region* and the other variable. For all models of [Hb], p values for *transmission region* were <0.0001, except for with the variable *Log_10_ parasite density* and *axillary temperature* for which was p=0.091 and p=0.022 respectively. For all anaemia models, overall p values for *transmission region* were <0.0001 except for the variables *village* and *Log_10_parasite density*, for which p was 0.013 and 0.0072 respectively. P values less than 0.05 are in bold.
